# Brimonidine Beyond a Single Specialty: Pharmacological Profile, Dermatologic Applications, and Advances in Drug Delivery Systems

**DOI:** 10.3390/ijms27031281

**Published:** 2026-01-27

**Authors:** Weronika Jóźwiak, Małgorzata Pietrusiewicz, Magdalena Piechota-Urbańska, Magdalena Markowicz-Piasecka

**Affiliations:** 1 Students Research Group, Department of Applied Pharmacy, Faculty of Pharmacy, Medical University of Lodz, Muszyńskiego 1, 90-151 Łódz, Poland; weronika.miruk@stud.umed.lodz.pl; 2 Department of Applied Pharmacy, Faculty of Pharmacy, Medical University of Lodz, Muszyńskiego 1, 90-151 Łódz, Poland; malgorzata.pietrusiewicz@umed.lodz.pl (M.P.); magdalena.piechota-urbanska@umed.lodz.pl (M.P.-U.)

**Keywords:** brimonidine, dermatology, α-adrenergic agonist, erythema

## Abstract

Brimonidine, a highly selective α_2_-adrenergic receptor agonist originally developed for glaucoma treatment, has emerged as an important dermatological agent due to its potent vasoconstrictive and anti-inflammatory properties. This review summarizes its pharmacological characteristics, and clinical applications. By activating α_2_-adrenergic receptors in cutaneous vessels, brimonidine induces rapid, reversible vasoconstriction and reduces neurogenic inflammation, leading to significant improvement of facial erythema in rosacea. Beyond its approved indication, topical brimonidine demonstrates efficacy in alcohol flushing syndrome, telangiectasia, post-procedural erythema, and as a local hemostatic agent in dermatologic surgery. Its favorable safety profile and minimal systemic absorption make it suitable for long-term use, though transient rebound erythema may occur. Advances in nanotechnology—such as supramolecular hydrogels and lipid-based carriers—enhance skin retention, prolong therapeutic action, and improve tolerability. These developments, together with ongoing synthesis of new quinoxaline–imidazoline analogues, open prospects for next-generation α_2_-agonists with optimized selectivity and dermatologic applicability. Brimonidine’s emerging role extends to dermatologic formulations for transient redness and sensitive skin management. Integrating pharmacological, formulation, and molecular insights may transform brimonidine from a niche rosacea therapy into a versatile platform for vascular, inflammatory, and aesthetic skin treatments.

## 1. Introduction

Over the past two decades, dermatology has increasingly adopted the practice of drug repurposing, including both off-label prescribing and the cosmetic adaptation of pharmaceutical agents. This approach has become an important driver of therapeutic innovation, particularly in conditions where approved treatments are limited or where rapid symptom control is required [1,2]. In dermatology, this is a common and often evidence-supported approach due to the evolving nature of skin-related disorders and therapeutic needs [3]. In dermatology, several medications originally developed for other conditions have found valuable off-label applications. One well-known example is minoxidil, initially formulated as an oral treatment for high blood pressure. It was later discovered that a side effect—excessive hair growth—could be harnessed therapeutically [4]. As a result, low-dose oral minoxidil is now used off-label to treat various types of hair loss beyond the approved topical indication for androgenetic alopecia [4]. α-Adrenergic agonists have become increasingly important in dermatology due to their ability to induce targeted vasoconstriction through activation of cutaneous smooth-muscle α-receptors. Topically applied α_1_-agonists, such as oxymetazoline, constrict superficial dermal vessels and are approved for persistent facial erythema in rosacea, offering sustained reduction in redness with favorable tolerability profiles [5,6,7].

Brimonidine tartrate represents another successful transition of a systemic or ophthalmologic drug into dermatological and aesthetic use. This drug was first approved for the treatment of open-angle glaucoma and ocular hypertension [8]. By decreasing aqueous humor production and enhancing uveoscleral outflow, it effectively reduces intraocular pressure [9]. Beyond ophthalmology, brimonidine has gained attention in dermatology due to its vasoconstrictive properties, which have led to its off-label and approved use in managing facial erythema associated with rosacea [8]. Apart from this indication, brimonidine has shown potential in several off-label dermatological applications. Due to its potent vasoconstrictive and anti-inflammatory properties, it is being investigated for use in conditions characterized by vascular hyperreactivity, such as flushing beyond rosacea, post-procedural erythema (e.g., after laser therapy), alcohol flushing syndrome (AFS), and even facial blushing associated with social anxiety [8,10,11]. Additionally, preliminary studies have explored its use in infantile hemangiomas as a topical alternative to systemic β-blockers, though data are still limited [11].

Given the widespread practice of off-label prescribing in dermatology, expanding interest in drug repurposing for skin conditions, lack of consolidated reviews on non-rosacea dermatologic applications of brimonidine, need to clarify mechanisms of action, efficacy, and safety in these newer contexts, a dedicated review would fill a critical knowledge gap and serve as a valuable position for dermatologists, researchers, and cosmetic scientists. This review aims to provide a comprehensive and critical evaluation of brimonidine and related α2-adrenergic receptor agonists in dermatological applications. The scope of this publication includes: mechanistic insights into the pharmacological action of brimonidine in the skin, with a focus on vasoconstriction mediated by α2-adrenergic receptor activation, an overview of the safety profile and tolerability of brimonidine in chronic dermatologic use, exploration of potential cosmetic and aesthetic uses, formulation challenges for effective and stable topical delivery of brimonidine. In addition, we present nanotechnology-based strategies and advanced drug delivery systems aimed at enhancing skin targeting, prolonging effects, and improving user tolerability. As well as examine structure–activity relationships (SAR) of brimonidine and its analogs.

## 2. Methodology

The preparation of this review was based on a comprehensive and structured literature search performed between June and October 2025. To ensure a broad overview of brimonidine and its applications, several major scientific databases were consulted, including PubMed/MEDLINE, ScienceDirect, ACS Publications, Scopus, and Google Scholar. Searches were conducted without restriction on publication year to include both early foundational studies and the most recent advances. The search process was iterative: initial searches generated a core set of relevant publications, and additional articles were identified through forward and backward citation tracking.

During the literature search, numerous keyword combinations were employed to capture the multifaceted nature of brimonidine. These included terms such as “brimonidine + dermatology,” “brimonidine + erythema,” “brimonidine + adverse reactions,” “brimonidine + pharmacokinetics,” “brimonidine + nanotechnology,” “brimonidine + cosmetics,” “brimonidine drug delivery systems,” “brimonidine analogues,” “brimonidine derivatives,” “brimonidine + imidazole derivatives,” and “brimonidine + quinoxaline derivatives.” Additional keywords were added throughout the process when new research directions emerged, allowing the search strategy to remain flexible and comprehensive.

All article types relevant to the objectives of this review were considered, including experimental studies, clinical trials, pharmacokinetic analyses, case reports, and mechanistic investigations. We also evaluated the reliability of the cited studies by considering their design (randomized, controlled, observational, or mechanistic), sample size, methodological transparency, and reproducibility of outcomes. When interpreting individual findings, we prioritized data derived from well-designed clinical trials and regulatory documents, while clearly acknowledging the limitations of preliminary or small-sample studies. Furthermore, previously published narrative and systematic reviews available in PubMed were consulted to ensure thorough background coverage and to verify that no essential publications were overlooked. Only articles written in English and presenting verifiable scientific data were included.

To complement scientific literature, regulatory and product-specific documents were also examined. In particular, materials from the European Medicines Agency (EMA) pertaining to brimonidine and its dermatological use were reviewed, along with official product characteristics of Mirvaso^®^ (brimonidine 0.33% gel). These documents, listed in the bibliography of the manuscript, provided essential information on approved indications, dosing recommendations, safety warnings, pharmacovigilance reports, and regulatory assessments relevant to the clinical use of brimonidine. Together, these sources formed the evidence base for the review and allowed for a comprehensive, multidisciplinary discussion of the pharmacology, clinical utility, safety, and formulation strategies associated with brimonidine.

## 3. Brimonidine—Basic Information

**Figure 1:**
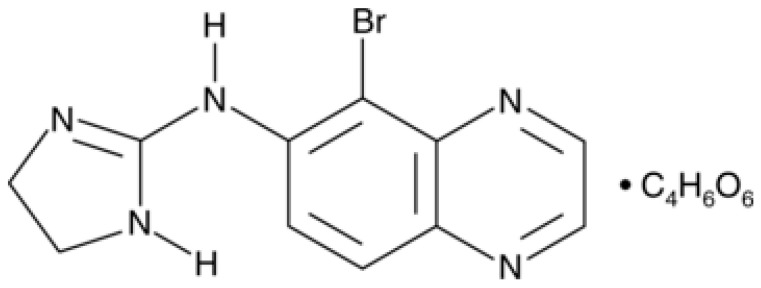
Chemical formula of brimonidine tartrate.

Brimonidine, chemically known as 5-bromo-6-(2-imidazolidinylideneamino)quinoxaline, is a highly selective alpha-2 adrenergic receptor agonist belonging to the class of 2-imidazoline derivatives. Its molecular formula is C_11_H_10_BrN_5_, with a molecular weight of 292.13 g/mol. The compound contains a quinoxaline ring system substituted with a bromine atom and an imidazoline group (Figure 1), structural features essential for its alpha-2 adrenergic activity. The structure of brimonidine is presented in Figure 1. First introduced in 1996 [12], brimonidine is regarded as a third-generation alpha-2 adrenergic receptor agonist, characterized by a markedly higher affinity for alpha-2 adrenoceptors compared to alpha-1 receptors [13,14,15]. Notably, it exhibits superior selectivity for alpha-2 adrenergic receptors relative to earlier agents such as clonidine or apraclonidine, with approximately 1000-fold greater selectivity for alpha-2 over alpha-1 receptors [16].

## 4. Pharmacological Activity in Ophthalmology

**Figure 2:**
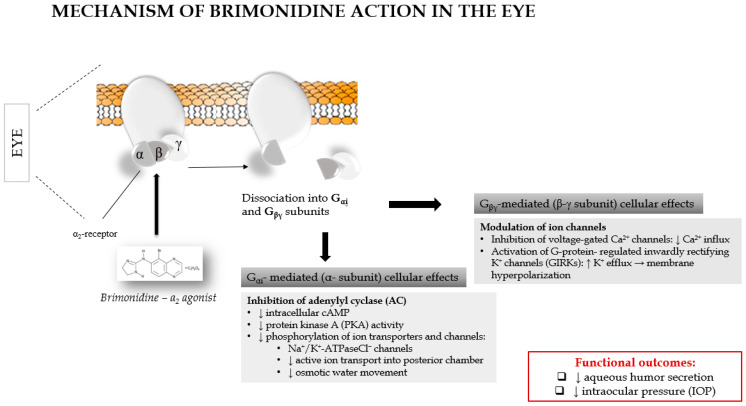
Brimonidine is a medication prescribed for the management of open-angle glaucoma and ocular hypertension. The illustration depicts its molecular mechanism of action in the ciliary epithelium. In essence, brimonidine reduces the production of aqueous humor while simultaneously enhancing its drainage from the eye via the uveoscleral outflow pathway (↑—increase, ↓—decrease).

The ophthalmic efficacy of brimonidine arises from a dual, receptor-mediated molecular mechanism involving both suppression of aqueous humor production and enhancement of uveoscleral outflow (Figure 2) [9]. At the molecular level, brimonidine selectively activates α_2_-adrenergic receptors (predominantly the α_2_A subtype) expressed on non-pigmented ciliary epithelial cells and uveoscleral pathway-associated tissues. Receptor activation triggers Gi protein coupling, leading to inhibition of adenylyl cyclase, a reduction in intracellular cAMP levels, and subsequent downregulation of protein kinase A (PKA) activity. This signaling cascade decreases ion transport and secretory activity in ciliary epithelial cells, thereby reducing aqueous humor formation.

Concomitantly, α_2_-adrenergic receptor activation modulates intracellular signaling pathways that influence cytoskeletal organization and extracellular matrix remodeling, facilitating increased uveoscleral outflow. Together, these molecular events result in a sustained reduction of intraocular pressure. A schematic representation of the molecular signaling pathways underlying brimonidine action in ocular tissues is presented in Figure 2.

Beyond its pressure-lowering effects, brimonidine exhibits favorable molecular stability, characterized by resistance to oxidative degradation. This chemical property reduces the formation of reactive intermediates that may trigger immune responses, contributing at the molecular level to a lower incidence of ocular allergic reactions compared with other α_2_-adrenergic receptor agonists [17].

From a translational perspective, the first ophthalmic formulation of brimonidine was approved by the U.S. Food and Drug Administration (FDA) in 1996 under the trade name Alphagan [18], and it remains the only highly selective α_2_-adrenergic receptor agonist approved for long-term glaucoma therapy [9]. In clinical practice, brimonidine is also available as a fixed-dose combination with the carbonic anhydrase inhibitor brinzolamide (Simbrinza), enabling additive intraocular pressure reduction through complementary molecular mechanisms—Gi-mediated suppression of aqueous humor production and inhibition of bicarbonate-dependent fluid secretion [19].

Although its clinical significance has not yet been fully established, experimental evidence indicates that brimonidine may also exert neuroprotective effects in models of cerebral ischemia and optic nerve injury [20]. In vitro studies further demonstrate that brimonidine protects neuronal cells against kainic acid-induced toxicity and preserves cultured retinal ganglion cells from glutamate-mediated cytotoxicity, a process implicated in secondary neuronal degeneration in glaucoma [21].

Neuroprotective effects of brimonidine have also been demonstrated in rat models of acute retinal ischemia and chronic intraocular pressure (IOP) elevation [22]. It has been proposed that these actions may be mediated through the enhancement of intrinsic retinal ganglion cell survival mechanisms and/or the induction of neuronal survival factors, such as basic fibroblast growth factor (bFGF) [23]. While these findings are promising, the clinical significance of brimonidine’s neuroprotective properties remains to be confirmed, and further investigations are required to establish their therapeutic relevance.

The most commonly reported adverse reactions to ophthalmic brimonidine include ocular dryness, itching, redness, and blurred vision [24]. In addition, some patients may experience allergic conjunctivitis, follicular conjunctivitis, or nonspecific ocular inflammation, which are generally associated with long-term therapy [18,25,26,27]. These adverse events are typically mild to moderate in severity, although allergic reactions may necessitate discontinuation of treatment in a subset of patients. The most common systemic side-effects are oral dryness and fatigue or drowsiness [9].

## 5. Pharmacokinetic Properties of Brimonidine

Brimonidine readily penetrates the cornea after ocular administration, reaching pharmacologically relevant concentrations in the aqueous humor and ciliary body, the primary sites of its intraocular pressure-lowering effects [9]. Following ocular instillation of 0.2% brimonidine solution, peak plasma concentrations (C_max_) are achieved within 1–4 h [12]. In a clinical study involving repeated cutaneous application for the treatment of facial erythema of rosacea, systemic exposure was minimal, with no evidence of drug accumulation. Reported values for systemic exposure were C_max_ = 46 ± 62 pg/mL and AUC = 417 ± 264 pg·h/mL [16].

The volume of distribution of brimonidine in humans has not been established. In preclinical studies, brimonidine was shown to cross the placenta and enter the fetal circulation to a limited extent [12]. Due to its relatively low lipophilicity, the compound is not expected to cross the blood–brain barrier to a significant degree [12]. Data on plasma protein binding of brimonidine are currently unavailable [16].

Evidence suggests that brimonidine undergoes initial metabolism in the cornea. Following systemic absorption after topical administration, the drug is extensively metabolized in the liver, primarily through pathways mediated by hepatic aldehyde oxidases [12]. Brimonidine and its metabolites are predominantly excreted in the urine, with approximately 74% of the administered dose recovered. The systemic elimination half-life following ocular administration of 0.2% solution is approximately 3 h [12]. The exemplary PK studies of brimonidine are summarized in Table 1.

## 6. Brimonidine in Dermatology—Mechanism of Action and Efficacy

Preparations containing brimonidine, most commonly in the form of a 0.33% gel, exert a potent vasoconstrictive effect that reduces facial erythema, one of the most distressing symptoms of rosacea. Brimonidine tartrate acts locally by selectively stimulating α_2_-adrenergic receptors expressed on vascular smooth muscle cells within the superficial dermis. Activation of these receptors triggers Gi protein-coupled signaling pathways, leading to a decrease in intracellular cAMP levels, smooth muscle contraction, and consequent narrowing of cutaneous blood vessels [31,32,33]. The vasoconstrictive effect typically manifests within 30–60 min after application and persists for approximately 9–12 h, substantially diminishing redness and improving patients’ quality of life and social functioning.

**Figure 3:**
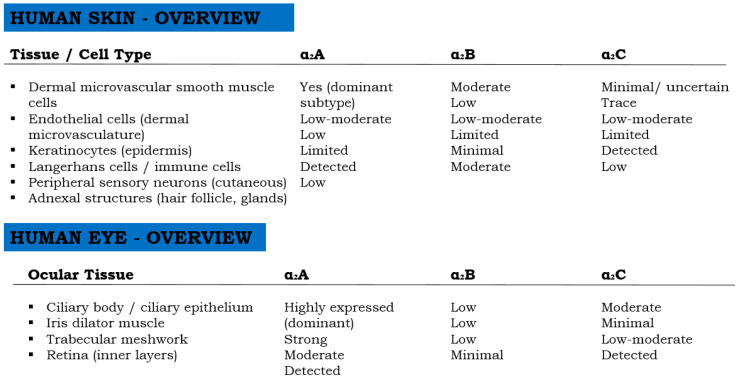
α_2_-Adrenergic Receptor Subtype Distribution (α_2_A, α_2_B, α_2_C).

At the molecular level, α_2_-adrenergic receptors are classified into three subtypes—α_2_A, α_2_B, and α_2_C—each with distinct tissue distributions and signaling properties that contribute to differential pharmacological effects in the skin versus ocular tissues (Figure 3). In human skin, α_2_A receptors predominate in the cutaneous microvasculature, including the smooth muscle cells of arterioles and venules, mediating sustained vasoconstriction. α_2_B receptors are primarily involved in transient vasoconstrictive responses, whereas α_2_C receptors are located in perivascular sympathetic nerve terminals and may regulate neurotransmitter release [34,35,36,37]. A schematic illustration of α_2_-adrenergic receptor subtype distribution is presented in Figure 3, highlighting the molecular basis of brimonidine’s localized vascular effects.

Dermatological studies on brimonidine differ in methodological rigor; therefore, where relevant, study design and the robustness of conclusions are critically discussed to provide a reliable overview of the evidence.

In contrast, the eye shows abundant expression of α_2_A receptors in the ciliary epithelium, trabecular meshwork, and retina, mediating both vasoconstriction and reduced aqueous humor production, while α_2_C receptors contribute to neuroprotective signaling pathways [38].

Brimonidine exerts its pharmacological effects through a well-defined molecular signaling cascade mediated by α_2_-adrenergic receptors, which are G protein-coupled receptors (GPCRs) primarily coupled to Gi/o proteins. Upon ligand binding, activation of these receptors leads to inhibition of adenylate cyclase, resulting in a reduction of intracellular cyclic adenosine monophosphate (cAMP) levels and subsequent downregulation of protein kinase A (PKA) activity.

At the molecular level, decreased PKA activity alters the phosphorylation state of key contractile regulators within vascular smooth muscle cells. In particular, reduced PKA-mediated phosphorylation shifts the regulatory balance toward activation of myosin light chain kinase (MLCK), promoting phosphorylation of myosin light chains. This molecular event enhances actin–myosin interactions, leading to smooth muscle contraction and vasoconstriction of superficial arterioles and venules [39]. Clinically, this translates into a rapid and visible reduction in erythema following topical brimonidine application.

Importantly, brimonidine demonstrates high selectivity for post-synaptic α_2_A-adrenergic receptors expressed on endothelial and vascular smooth muscle cells. This receptor subtype specificity underlies its potent vasoconstrictive action in human subcutaneous blood vessels with diameters smaller than 200 µm. Furthermore, brimonidine has been shown to inhibit capsaicin-induced vasodilation, indicating interference with neurogenic inflammatory signaling pathways and supporting its role in modulating sensory nerve-mediated vascular responses [40].

**Figure 4:**
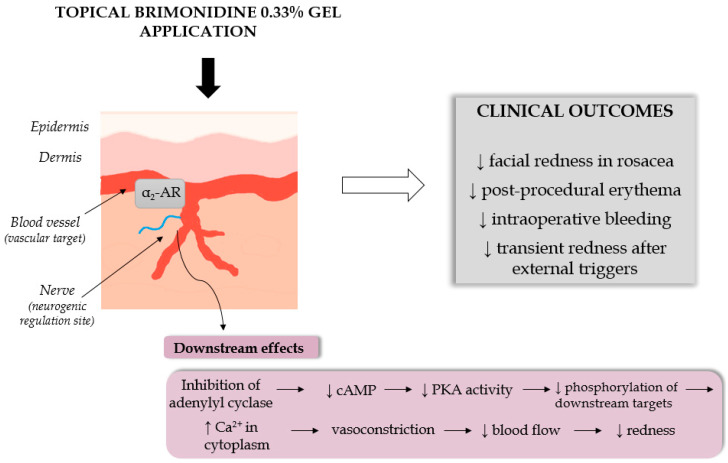
Mechanism of Brimonidine Action in Cutaneous Vasculature and Inflammation. ↑—increase, ↓—decrease, AR—adrenergic receptor.

Figure 4 schematically illustrates the molecular mechanism of brimonidine action in the skin, highlighting receptor activation, Gi/o protein coupling, downstream inhibition of the cAMP–PKA pathway, and MLCK-driven vasoconstriction.

Beyond the canonical Gi protein-mediated inhibition of adenylyl cyclase and subsequent suppression of the cAMP/PKA axis, activation of α_2_-adrenergic receptors by brimonidine initiates multiple intracellular molecular signaling pathways that are highly relevant to cutaneous physiology. These non-canonical signaling routes include activation of the MAPK/ERK pathway, engagement of phospholipase C (PLC) with downstream inositol-1,4,5-trisphosphate (IP_3_)-dependent Ca^2+^ mobilization, stimulation of the PI3K/Akt signaling cascade, and Rho-kinase-dependent regulation of cytoskeletal dynamics and vascular smooth muscle contractility [41].

At the molecular level, these pathways converge to modulate vascular tone, inflammatory signaling, endothelial permeability, and neurovascular coupling. MAPK/ERK and PI3K/Akt signaling influence gene transcription and inflammatory mediator production, while PLC–IP_3_/Ca^2+^ signaling and Rho-kinase activation directly regulate actomyosin contraction and endothelial barrier integrity. Together, these molecular events complement the Gi–cAMP axis and fine-tune the vasoconstrictive and anti-inflammatory responses induced by brimonidine in the skin.

Importantly, tissue-specific differences in α_2_-adrenergic receptor subtype expression (α_2_A, α_2_B, α_2_C) and downstream effector coupling underlie divergent molecular outcomes. In ocular tissues, dominance of α_2_A-mediated Gi–cAMP signaling results in robust vasoconstriction and sustained suppression of aqueous humor production. In contrast, the heterogeneous receptor subtype distribution and broader engagement of auxiliary signaling pathways in the skin lead to attenuated and short-lived vasoconstriction, accompanied by anti-inflammatory and neurovascular modulatory effects [42,43].

This molecular divergence in receptor signaling architecture and intracellular pathway engagement provides a mechanistic explanation for the prolonged intraocular efficacy of brimonidine versus its transient erythema-reducing effect in dermatologic applications [44].

At the neurovascular level, activation of presynaptic α_2_-receptors on sympathetic nerve terminals inhibits norepinephrine release, thereby attenuating neurogenic vasodilation. Brimonidine may also influence transient receptor potential (TRP) channels—notably TRPV1 and TRPA1—on sensory nerve endings, decreasing neuropeptide release (e.g., substance P, calcitonin gene-related peptide (CGRP)) and vascular hyperreactivity [45,46]. Furthermore, the anti-inflammatory properties of brimonidine in the skin have been confirmed in murine ear inflammation models, where treatment with brimonidine significantly reduced experimentally induced ear edema compared with the corresponding vehicle control. Experimental data indicate that α_2_-receptor stimulation reduces vascular permeability and leukocyte adhesion, as well as inflammatory cytokine release (e.g., tumor necrosis factor—alpha (TNF-α), interleukin 1β (IL-1β)) and reactive oxygen species (ROS) generation [47]. These actions contribute to endothelial stabilization and may mitigate the inflammatory component of rosacea. According to the study of Bertino et al. [48] brimonidine exhibits notable anti-inflammatory activity in addition to its well-characterized vasoconstrictive effects. Experimental studies have shown that brimonidine modulates vascular barrier function and reduces leukocyte recruitment during inflammatory responses [48]. In vitro, it inhibits neutrophil transmigration across the endothelial barrier by regulating cell adhesion molecules, while in vivo murine models confirm that topical brimonidine prevents leukocyte rolling and adhesion in inflamed skin. Furthermore, clinical data demonstrate that pretreatment with brimonidine tartrate 0.33% gel significantly reduces UV-induced neutrophil infiltration in human skin by approximately 54%, supporting its ability to attenuate acute cutaneous inflammation [48].

Collectively, these findings demonstrate that brimonidine exerts anti-inflammatory effects through defined molecular mechanisms downstream of α_2_-adrenergic receptor activation, extending its therapeutic action beyond simple vasoconstriction. At the neurovascular level, brimonidine interferes with neurogenic inflammatory signaling, likely by attenuating sensory nerve-mediated release of pro-inflammatory neuropeptides, while concurrently suppressing neutrophil recruitment through modulation of endothelial activation and chemotactic signaling pathways.

Importantly, brimonidine exhibits direct immunomodulatory effects mediated at the molecular and transcriptional levels. In an LL-37-induced rosacea-like mouse model, topical application of brimonidine gel significantly improved clinical signs of inflammation and erythema [49]. Histological examination revealed a pronounced reduction in inflammatory cell infiltration, particularly mast cells, which are central effector cells in rosacea pathogenesis. At the molecular level, brimonidine treatment significantly downregulated mRNA expression of mast-cell-specific enzymes and inflammatory mediators that were otherwise upregulated by LL-37, indicating suppression of mast cell activation, degranulation pathways, and cytokine signaling networks [49].

**Figure 5:**
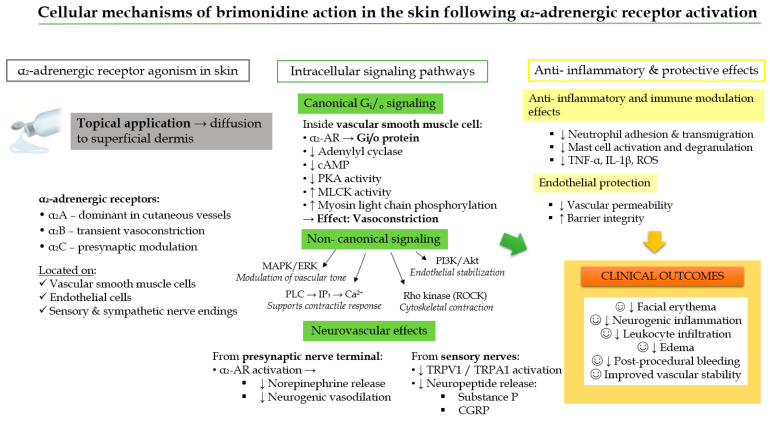
Cellular mechanisms of brimonidine action in the skin (↑—increase, ↓—decrease).

These molecular findings suggest that brimonidine not only alleviates vascular symptoms via α_2_-adrenergic receptor–Gi protein signaling, but also modulates immune cell function through regulation of gene expression and inflammatory signaling cascades. Such dual activity—combining vascular and immunological molecular mechanisms—contributes to the overall therapeutic efficacy of brimonidine in rosacea. A schematic summary of brimonidine’s molecular, vascular, and immunomodulatory actions in the skin is presented in Figure 5.

Brimonidine activates α_2_-adrenergic receptors (α_2_A/α_2_B/α_2_C) expressed on vascular smooth muscle cells, endothelial cells, and presynaptic nerve terminals. Receptor coupling to Gi/o proteins inhibits adenylyl cyclase, reduces cAMP/PKA signaling, and promotes myosin light chain kinase-mediated contraction, resulting in vasoconstriction of superficial dermal vessels. Concurrent modulation of MAPK, PI3K/Akt, PLC–IP_3_/Ca^2+^, and Rho-kinase pathways further stabilizes vascular tone and endothelial integrity. Presynaptic α_2_-receptor activation suppresses norepinephrine release and attenuates neurogenic inflammation by reducing TRP-channel-mediated neuropeptide secretion. In addition, brimonidine decreases leukocyte adhesion and transmigration, mast cell activation, pro-inflammatory cytokine release, and oxidative stress, contributing to its anti-inflammatory and protective effects in erythematous and vascular skin disorders.

Brimonidine has been shown to exhibit therapeutic efficacy across a range of dermatological indications, including rosacea, erythema, post-surgical hemostasis, telangiectasia, and alcohol flushing syndrome (AFS). Topical formulations of brimonidine are currently approved for the management of facial erythema associated with rosacea and have been further investigated for their potential utility in minimizing post-procedural bleeding and enhancing outcomes in vascular lesions.

A comprehensive review of the current literature [8] confirms that brimonidine effectively mitigates symptoms of rosacea, erythema, post-surgical bleeding, telangiectasia, and AFS. When administered topically prior to surgical procedures, brimonidine may contribute to improved intra- and postoperative hemostasis by reducing intraoperative bleeding and diminishing the requirement for cauterization. Moreover, topical brimonidine has been recognized as a valuable therapeutic option for telangiectasia, and it may additionally alleviate erythema and discomfort following intense pulsed light (IPL) therapy [33].

Erythema associated with rosacea results from vasomotor dysregulation leading to persistent peripheral arterial dilation, which poses a significant therapeutic challenge. Owing to its potent vasoconstrictive properties, brimonidine has been employed in the management of rosacea-related erythema. In a clinical study, Fowler et al. [50] evaluated the efficacy of topical brimonidine tartrate (BT) gel at concentrations of 0.07%, 0.18%, and 0.5% in patients with moderate to severe rosacea. The degree of facial erythema was assessed using both the Clinician’s Erythema Assessment (CEA) and the Patient’s Self-Assessment (PSA) scales. Significant improvements—defined as a two-grade reduction in erythema severity—were observed on days 1, 15, and 29 across treatment groups, with the 0.5% BT gel demonstrating the highest response rate compared with the vehicle control. Among the tested concentrations, the 0.5% formulation yielded the most favorable therapeutic outcomes [50]. The findings from Fowler et al. [50] are supported by a robust randomized, vehicle-controlled design with adequate sample size, which increases the reliability of the observed dose-dependent improvements in erythema.

In a separate clinical trial, participants applied 0.5% BT gel once daily for four weeks. The Patient’s Assessment of Appearance (PAA) scale was used to evaluate overall skin appearance, with scores ranging from 0 (“extremely satisfied”) to 4 (“very dissatisfied”). Eighty percent of patients demonstrated a two-grade improvement in erythema severity on both the PSA and Clinician’s Erythema Assessment (CEA) scales and reported higher satisfaction on the PAA scale. A strong positive correlation between PAA and CEA/PSA scores was observed on days 1, 15, and 29 [51]. Although the study employed validated scales and demonstrated consistent clinician- and patient-reported outcomes, the relatively short treatment duration is a limitation that should be considered when interpreting the long-term implications. Similarly, evaluation of 0.33% brimonidine gel in patients with erythematotelangiectatic rosacea characterized by persistent background erythema and visible telangiectasia produced comparable results. Erythema was markedly reduced within six hours of treatment initiation; however, no significant improvement in telangiectasia was observed [51].

Micali et al. [52] further evaluated a combined therapeutic approach using brimonidine and Nd:YAG laser to target erythema and telangiectasia, respectively. Treatment efficacy was assessed using the Investigator’s Global Assessment (IGA), a five-point rating scale ranging from “absent” to “outstanding.” Following Nd:YAG laser therapy, facial erythema persisted, although the extent of telangiectasia was reduced. When both brimonidine gel and Nd:YAG laser were applied concomitantly, a notably improved IGA score was achieved, suggesting that combined therapy enhances clinical outcomes in patients with rosacea. Because this was a combination-treatment study without long-term follow-up, the conclusions should be interpreted cautiously, although the split-modality approach provides internally controlled comparative data.

Recent clinical investigations have also explored inclusion brimonidine in combination regimens—for instance, with tranexamic acid (TXA) and oxymetazoline (OXZ)—to enhance therapeutic outcomes in conditions such as persistent post-acne erythema (PAE) [53]. In a split-face comparative study, a topical formulation containing TXA 5%, OXZ 1.5%, and BM 0.33% demonstrated a significant reduction in erythema compared with placebo, confirming the synergistic benefits of combining vasoconstrictive and antifibrinolytic agents. These findings highlight the expanding clinical utility of brimonidine as a targeted and safe approach for the management of various erythematous skin conditions [53].

In another clinical evaluation, once-daily application of brimonidine gel 0.33% demonstrated significant efficacy in reducing facial erythema in patients with rosacea who perceived their redness as severe [54]. After eight days of treatment, a markedly greater proportion of subjects treated with brimonidine reported satisfaction with their facial appearance, overall treatment effect, and improvement in redness compared with those receiving vehicle (all *p* < 0.05). Objective assessments supported these findings, with significantly more patients achieving at least a one-grade improvement from baseline in both the Clinician’s Erythema Assessment (71.7% vs. 35.7%; *p* = 0.0011) and the Patient’s Self-Assessment scores (76.1% vs. 47.6%; *p* = 0.004). Furthermore, a higher percentage of patients in the brimonidine group were able to maintain daily control of facial redness (83.0% vs. 38.9% on Day 1). Although mild and transient adverse events were more frequent with brimonidine (29.2% vs. 15.9%), the treatment was generally well tolerated. Overall, brimonidine gel 0.33% provided rapid and sustained improvement in erythema control and patient satisfaction, underscoring its efficacy and safety as the first-line topical therapy for persistent facial erythema in rosacea [54].

Another important results of application of brimonidine in dermatology were presented by Wesley Yu et al. [55]. In a randomized, double-blind, placebo-controlled split-face clinical trial, they evaluated the efficacy of topical brimonidine gel 0.33% in reducing alcohol-induced facial erythema in individuals with AFS. Twenty healthy volunteers of East Asian descent with self-reported AFS applied brimonidine gel to one half of the face and placebo to the other prior to alcohol consumption. Sixty minutes after application, facial erythema was significantly reduced on the brimonidine-treated side, with mean decreases of 2.1 and 1.7 points in Clinician Erythema Assessment and Subject Self-Assessment scores, respectively (both *p* < 0.001). The effect persisted for up to 120 min post-application. Participants also reported a high likelihood of using the treatment again and recommending it to others. These findings demonstrate that brimonidine gel 0.33% provides a rapid and clinically meaningful reduction of alcohol-induced erythema in AFS and may offer a therapeutic option for patients experiencing psychosocial distress related to facial flushing [55]. While the study was randomized and double-blind, the relatively small number of participants means the conclusions, although promising, should be considered preliminary.

Another dermatological use of brimonidine was presented by Chen et al. [56]. In a randomized pilot study evaluating its hemostatic potential during Mohs micrographic surgery (MMS), topical brimonidine 0.33% gel significantly reduced intraoperative bleeding in patients receiving anticoagulant therapy. Fourteen patients received preoperative brimonidine application, while ten served as controls. Compared with standard care, the brimonidine-treated group exhibited a 68% reduction in blood loss over 30 s (*p* < 0.05) and required markedly less electrocautery—none of the treated patients required cauterization of more than half of the wound bed, compared with 80% in the control group. In addition, erythema at the surgical site was nearly four times lower in the brimonidine group (*p* < 0.01). These findings demonstrate that preoperative use of topical brimonidine gel provides effective local vasoconstriction, substantially decreasing bleeding and surgical field erythema during MMS, particularly in patients on anticoagulant therapy [56]. However, the readers should keep in mind that the pilot nature of this study and its limited group size reduce the overall strength of evidence. Complementing these findings, Lipner [57] reported the successful use of brimonidine 0.33% gel in ten nail surgery procedures, where topical application achieved rapid hemostasis within 20–30 s without adverse effects or delayed wound healing. Together, these studies highlight the emerging utility of topical brimonidine as a safe, effective, and non-destructive adjunct for controlling bleeding in cutaneous surgical settings.

There are also other examples of effective use of brimonidine in dermatological conditions. For instance, in a randomized, single-blinded, split-face clinical trial involving 19 patients with moderate to severe facial telangiectasias, the effects of topical brimonidine 0.33% gel applied after intense pulsed light (IPL) treatment were evaluated against air-cooling alone [58]. The adjuvant use of brimonidine significantly reduced post-IPL erythema, restoring redness to baseline levels within 24 h and achieving a median erythema reduction of 50–95% compared with 9–28% on the control side (*p* = 0.002). Although no difference in edema reduction was observed, brimonidine consistently decreased postoperative pain (VAS 1.0 vs. 1.5–2.0, *p* = 0.032). Both facial sides demonstrated excellent clearance of telangiectasias (75–100%), indicating that brimonidine did not interfere with IPL efficacy. Moreover, 79% of patients preferred the brimonidine-treated side (*p* = 0.019). Overall, topical brimonidine effectively minimized IPL-induced erythema and discomfort while maintaining high treatment efficacy, confirming its value as an adjuvant therapy for facial telangiectasias [58]. This split-face design offers a high level of internal control, but the small sample size limits the generalizability of the results.

Despite consistent evidence that topical brimonidine reduces visible facial erythema, several mechanistic and clinical uncertainties remain. The cellular mediators of rebound erythema are not defined, and the relative contribution of vasoconstriction versus anti-inflammatory actions to clinical benefit has not been dissected in humans. There is limited evidence that brimonidine improves fixed telangiectasia, and objective imaging studies linking vessel anatomy to response are lacking. Head-to-head comparisons versus other topical vasoconstrictors (e.g., oxymetazoline), data across diverse skin phototypes, and robust dermal PK/PD studies are also missing. Addressing these gaps would require mechanistic human studies (micro-dialysis, biomarkers), imaging-based responder analyses, and adequately powered comparative clinical trials.

**Figure 6:**
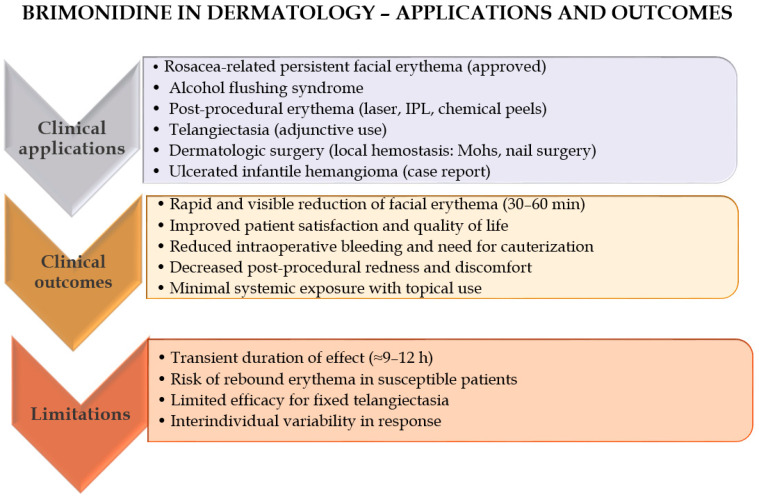
Brimonidine in dermatology—applications and outcomes.

In summary, BT effectively reduces facial erythema in rosacea by inducing rapid and sustained vasoconstriction, improving both clinician- and patient-reported outcomes. Applications and outcomes of brimonidine in dermatology have been summarized in Figure 6.

Beyond rosacea, brimonidine has demonstrated efficacy in alcohol flushing syndrome and as a hemostatic agent in Mohs micrographic and nail surgeries, significantly decreasing intraoperative bleeding and the need for cauterization. It also reduces post-procedural erythema and discomfort following intense pulsed light therapy for telangiectasias without compromising treatment efficacy. Combination regimens with agents such as TXA or OXY further enhance therapeutic outcomes in conditions like persistent post-acne erythema. Overall, topical brimonidine is a safe, rapid-acting, and versatile agent for managing erythematous and vascular skin conditions, as well as improving procedural and surgical outcomes. To address concerns regarding descriptive summarization, we present a comparative critical appraisal of representative dermatologic studies evaluating topical brimonidine (Table 2). This table highlights methodological strengths, weaknesses, sample size constraints, and the level of evidence supporting each indication.

**Figure 7:**
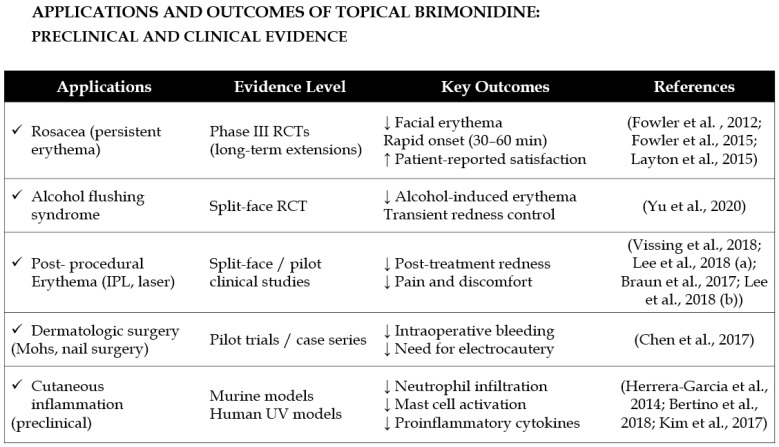
Applications and outcomes of topical brimonidine across dermatological indications. The figure summarizes clinical and preclinical applications of topical brimonidine, highlighting the level of supporting evidence and key therapeutic outcomes. Brimonidine demonstrates rapid vasoconstrictive, anti-inflammatory, and hemostatic effects, resulting in reduced erythema, bleeding, and post-procedural discomfort across multiple dermatological settings. Abbreviations: RCT—randomized controlled clinical trial. IPL—intense pulsed light therapy; ↑—increase, ↓—decrease. Citation: Fowler et al., 2012 [50]; Fowler et al., 2015 [51]; Layton et al., 2015 [54]; Yu et al., 2020 [55]; Vissing et al., 2018 [58]; Lee et al., 2018 (a) [61]; Braun et al., 2017 [62]; Lee et al., 2018 (b) [63]; Chen et al., 2017 [56]; Herrera-Garcia et al., 2014 [47]; Bertino et al., 2018 [48]; Kim et al., 2017 [49].

Applications, evidence level and outcomes of topical brimonidine is summarized in Figure 7.

## 7. Safety Profile and Tolerability in Chronic Skin Use

BT medications for topical use quickly gained recognition among dermatologists. In terms of safety, brimonidine tartrate has a favorable profile [8]. Both clinical studies and patients experience confirm the effectiveness and good tolerability of this substance administered topically. Most safety data derive from multiple clinical trials and extensive post-marketing surveillance, which provides a strong and reliable evidence base for the overall tolerability profile. However, case reports describing rebound erythema and contact dermatitis highlight individual variability and underscore the need for cautious interpretation of rare adverse reactions.

Based on extensive clinical trials and post-marketing reports, a small percentage of patients experienced adverse reactions after gel application. Most adverse reactions are cutaneous, mild to moderate, and usually resolve spontaneously with continued use or after discontinuation [33,54]. Side effects after topical use of brimonidine only affect the skin at the application site. Most frequently occurring adverse experiences were consistent with the safety profile of brimonidine and included redness, irritation, itching, burning, skin dryness or feeling of heat. Furthermore, some patients experienced contact dermatitis [64]. Interestingly enough, and paradoxically so, there have been cases of intensification of erythema after therapy following the doctor’s instructions. Isolated case reports describe rebound erythema occurring several hours after application, even under correct use. For instance, a young patient who applied 0.33% BT gel to her entire face every morning reported a recurrence of redness 12 h after application and even an increase in redness compared to the initial state before application. The mentioned redness was transient. While these accounts illustrate clinical variability, their generalizability is limited. In some cases, this leads to treatment discontinuation, but appropriate patient education and gradual introduction can often avoid this problem. It’s also worth noting that side effects usually resolve spontaneously after a few days or weeks of use [65]. Nevertheless, patients should be informed about possible adverse effects.

In the vast majority of cases, the benefits of treatment with brimonidine outweigh the risk of side effects. In a randomized, vehicle-controlled trial of 92 patients, 71.7% of subjects treated with brimonidine achieved at least a one-grade clinician-assessed improvement in erythema, and 76.1% reported at least a one-grade patient-assessed improvement after 8 days of treatment. Additionally, approximately 80% of patients reported successful daily control of facial redness during the study period [54]. A recent systematic review and meta-analysis published in Actas Dermo-Sifiliográficas [66] synthesized evidence from 21 randomized controlled trials evaluating commonly used topical therapies for rosacea. Within this comparative framework, brimonidine consistently demonstrated significant reductions in persistent facial erythema, with effect sizes aligned with those observed in pivotal phase III studies. The analysis also confirmed that brimonidine possesses a favorable short-term safety and tolerability profile, comparable to other vasoconstrictive agents such as oxymetazoline [66]. Importantly, by consolidating outcomes across multiple independent trials, this meta-analysis provides higher-level corroboration that brimonidine offers a clinically meaningful, rapid-onset improvement in erythema and reinforces its role as an evidence-based option for symptomatic management.

In clinical trials and long-term extension studies, BT demonstrated a favorable tolerability profile with once-daily use over many months, without evidence of cumulative toxicity or increasing adverse events. These findings are supported by post-marketing pharmacovigilance data, which similarly indicate that most treatment-emergent reactions are mild, transient, and localized to the application site. As with other vasoconstrictive agents, correct use is essential: the gel should be applied sparingly to intact, dry skin, typically once daily, to minimize irritation or exaggerated vasomotor responses. Brimonidine is not effective for the inflammatory component of rosacea, and therefore patients presenting with papules or pustules require concurrent therapy with established anti-inflammatory agents such as ivermectin, metronidazole, timolol, or azelaic acid [60,67]. When appropriately selected, such combination regimens can enhance overall disease control while maintaining good tolerability.

A retrospective analysis pooling data from the two pivotal Phase III trials and a 52-week open-label extension provides additional insight into the dermatological safety profile of topical brimonidine [68]. In this combined dataset, flushing and transient worsening of erythema were identified as the most common treatment-related events, occurring in 5.4% of subjects in the Phase III studies and 15.4% of subjects during long-term use. Importantly, these reactions were predominantly mild to moderate in intensity, typically appeared early in the treatment course, and were short-lived, resolving either spontaneously or after treatment interruption. The authors concluded that although paradoxical erythema can occur, it is infrequent and generally self-limiting, further supporting the favorable and predictable tolerability profile of brimonidine gel in dermatological use [68].

Key safety uncertainties persist: the incidence, predictors and mechanism of rebound erythema are not well established; long-term safety in non-prescription use remains unstudied; pediatric safety, especially for infant or ulcerated skin, is based only on anecdote and case reports; and systemic exposure under maximal use or damaged-barrier conditions needs clearer quantification. Furthermore, the role of excipients in contact sensitization and the safety of combination topical regimens have not been systematically evaluated. Larger real-world safety registries, maximal-use PK studies, and controlled combination-safety trials are needed to close these gaps.

Because many safety observations originate from real-world use rather than controlled trials, they should be interpreted as supportive but not definitive; nonetheless, the consistency of findings across independent sources strengthens the reliability of the overall safety assessment. In conclusion, BT is a valuable tool in the treatment of rosacea-related redness. It is characterized by rapid onset, a favorable safety profile, and good tolerability with long-term use. Like any medication, it may cause side effects, but with proper use and appropriate dermatological care, most patients experience significant therapeutic benefits and improved quality of life.

## 8. Potential Applications in Cosmetology, e.g., Transient Erythema Control, Anti-Redness Creams

Although BT was originally used in ophthalmology to treat glaucoma and later in dermatology—primarily for the treatment of persistent redness in rosacea—there is currently growing interest in its potential applications in cosmetology [11,69]. Thanks to its ability to constrict blood vessels in the skin, brimonidine tartrate could potentially become a valuable ingredient in topical preparations, especially those intended for skin with vascular problems, sensitive skin, and skin prone to temporary redness. However, in the European Union, brimonidine cannot be classified or used as a cosmetic ingredient, as its primary mode of action is pharmacological—namely, receptor-mediated vasoconstriction via α_2_-adrenergic agonism. Under Regulation (EC) No 1223/2009, cosmetic ingredients may not exert pharmacological, immunological, or metabolic effects. Additionally, according to this document, cosmetic products are intended only to cleanse, perfume, change appearance, protect, or maintain the condition of the skin. Consequently, brimonidine is legally restricted to medicinal products only, and no brimonidine-containing cosmetic formulations are authorized or listed in the EU cosmetic ingredient database (CosIng). The only permitted topical brimonidine product in the EU is Mirvaso^®^ (brimonidine 0.33% gel), licensed as a prescription medicine for rosacea-related persistent erythema. Incorporating pharmacologically active substances into cosmetics blurs the boundary between cosmetics and medicines and may pose safety and legal challenges (Regulation (EC) No 1223/2009; EMA, 2019).

Reports describing “cosmetic” or “cosmetology” uses in the literature refer exclusively to off-label medical use, not to approved cosmetic products. Importantly, marketing or formulating brimonidine as a cosmetic would constitute the placing of a medicinal product on the market without authorization, carrying significant regulatory, legal, and safety implications. Furthermore, the risks of adverse reactions such as rebound erythema, contact dermatitis, and systemic absorption underscore the necessity for medical oversight and further support its classification as a pharmacologically active medicinal substance, not a cosmetic ingredient. Therefore, discussions of potential cosmetic applications must be framed as theoretical, investigational, or off-label medical concepts, rather than established or legally permissible cosmetic uses.

Although BT can theoretically reduce transient, environmentally induced facial redness—such as redness triggered by sudden temperature changes, stress, physical exertion, alcohol consumption, or spicy foods [70]—these effects relate to its pharmacological α_2_-adrenergic vasoconstrictor activity, and therefore fall under medicinal, not cosmetic, mechanisms in the European Union. In such situations, dilatation of superficial facial vessels contributes to visible redness; however, because brimonidine exerts a receptor-mediated action, its use for controlling transient erythema is restricted to off-label medical application, not cosmetic use. Any preparation containing brimonidine that aims to reduce redness would therefore be classified as a medicinal product, not a cosmetic, under EU Regulation (EC) No 1223/2009.

Another area in which BT has been explored—exclusively as an off-label medical intervention rather than a cosmetic ingredient—is the management of post-procedural erythema following aesthetic procedures such as laser therapy, chemical peels, or mesotherapy. These procedures often induce transient vascular redness that may be uncomfortable for patients. Clinical observations indicate that medically supervised application of brimonidine can reduce laser-related postoperative erythema through its pharmacological vasoconstrictor action; however, this use remains investigational and must be regarded as therapeutic rather than cosmetic, as the mechanism involves receptor-mediated vascular effects [61,62,63].

Several case reports have described the successful off-label medical use of BT in the management of ulceration associated with infantile hemangioma (IH). In these reports, a compounded topical preparation containing 0.2% brimonidine combined with 0.5% timolol—a non-selective β-blocker commonly used in IH—was applied twice daily for 7–10 days. All treated infants exhibited complete ulcer healing within this period, and no adverse reactions (topical or systemic) were reported [60]. While these findings suggest that the brimonidine–timolol combination may represent a promising therapeutic option for ulcerated IH, the evidence remains limited to small, uncontrolled case series. Consequently, its use should be regarded strictly as investigational medical therapy, and not as a cosmetic or routine dermatologic treatment.

It is important to emphasize that any topical use of brimonidine—particularly outside its approved indication—requires caution. Although generally well tolerated in single medical applications, brimonidine may cause rebound erythema, where redness intensifies once the vasoconstrictive effect subsides. Individual sensitivity varies, and patients with highly reactive skin may be especially prone to such effects [50]. While some exploratory studies have examined the potential of lower concentrations for non-medical contexts brimonidine cannot be used as a cosmetic ingredient under EU law, as its receptor-mediated action classifies it as a pharmacologically active substance. Any attempt to develop a brimonidine-containing cosmetic formulation would therefore fall under medicinal product regulation and would require full pharmaceutical development, including demonstration of safety, efficacy, and long-term tolerability in accordance with EU medicinal product legislation—not cosmetic regulations. The cosmetic uses of brimonidine described in the literature are based primarily on extrapolation from dermatological studies and a limited number of small clinical evaluations. As such, the reliability of their conclusions is low.

The following Table 3 summarizes theoretical or investigational applications discussed in the scientific literature. These do not represent approved cosmetic uses, and in the EU brimonidine cannot be included in cosmetic formulations due to its pharmacological mode of action.

## 9. Formulation Challenges and Strategies for Topical Delivery

Despite the clinical efficacy of brimonidine in dermatological conditions, several formulation-related challenges must be addressed to optimize safety, patient compliance, and therapeutic outcomes. These challenges span across areas such as skin tolerability, pharmacokinetics, chemical stability, and product aesthetics.

One of the foremost issues is achieving an appropriate balance between potency and tolerability. Brimonidine’s pharmacological action, while effective, may cause adverse cutaneous reactions when administered in excessively high concentrations or when formulated inappropriately. Commonly reported side effects include burning, stinging, pruritus, and in some cases, paradoxical erythema or rebound flare-ups. Such adverse events are most frequently observed during the initial days of treatment and are usually mild to moderate in intensity [71]. Additionally, clinical case reports have identified allergic contact dermatitis as a potential reaction to topical brimonidine gel, further emphasizing the importance of excipient selection and vehicle compatibility [72].

A critical factor influencing both tolerability and efficacy is the skin penetration profile of the formulation. As the site of action lies in superficial dermal vessels, the drug must traverse the stratum corneum without inducing skin barrier disruption. Formulations that facilitate too rapid or excessive absorption can not only increase irritation risk but may also lead to systemic absorption. Recent advancements in delivery technologies, including hydrogels, offer enhanced skin retention and more uniform distribution of the active compound. These systems may provide a controlled-release effect that reduces peak concentrations associated with irritation, while sustaining therapeutic levels over time. One study, for example, reported that a supramolecular hydrogel retained approximately three times more brimonidine in the skin compared to a commercial reference gel [73].

Another notable challenge is the occurrence of rebound erythema, a phenomenon where facial redness returns worse than baseline after the drug’s vasoconstrictive effect wears off. Although not fully understood mechanistically, it is believed to result from reactive vasodilation following sudden or intense vasoconstriction. This can be exacerbated by high concentrations, fast-onset vehicles, or uneven skin distribution. The literature reports both isolated cases and post-marketing analyses of this effect. For example, Ilkovitch and Pomerantz [62] described significant rebound erythema following long-term use of topical brimonidine, raising concern about compensatory vascular responses. Strategies to mitigate this issue include using lower-concentration formulations (0,33% instead of 0,5%), limiting the amount applied per area, and incorporating ingredients that delay or smooth onset of vasoconstriction [74].

From a chemical and physical stability standpoint, BT presents formulation challenges due to its sensitivity to oxidative degradation and pH extremes. A stability study found that exposure to 6% hydrogen peroxide at 40 °C for 24 h led to a reduction of over 50% in active drug content, indicating significant vulnerability under stress conditions [75]. Formulation strategies must therefore ensure antioxidative protection, stable pH buffering, and inert excipient selection. Furthermore, physical characteristics such as viscosity, homogeneity and spreadability must be maintained throughout the product’s shelf life to ensure dosing consistency and user acceptability.

While brimonidine is intended for local use, systemic exposure is possible under certain conditions, particularly with high-concentration products, frequent application, or compromised skin barrier function. In a pharmacokinetic study comparing systemic exposure from ophthalmic and dermal routes, measurable plasma levels of brimonidine were observed in subjects using topical gel under maximal use conditions [71]. Although systemic adverse events remain rare, the findings reinforce the importance of limiting percutaneous absorption through rational vehicle design and patient instruction.

Cosmetic acceptability represents a decisive factor in patient adherence to topical therapies, particularly in the management of facial conditions where comfort and appearance are paramount. As highlighted by Oliveira and Almeida [76] adherence rates to topical agents are frequently compromised by unfavorable sensory attributes such as greasiness, residue, or unpleasant odor, which can discourage consistent use and reduce treatment effectiveness. Formulations that are lightweight, non-sticky, rapidly absorbed, and easy to spread promote user satisfaction and long-term compliance. Conversely, unappealing textures or poor spreadability may lead to under- or over-application, unintentional contact with sensitive areas, and localized irritation. Therefore, optimizing the sensorial profile of a formulation—alongside pharmacological efficacy—is crucial for therapeutic success. Incorporating patient feedback, rheological assessment, and sensory testing during product development ensures that dermatological preparations achieve both functional performance and high cosmetic acceptability, ultimately improving real-world treatment outcomes [76]. The cosmetic uses of brimonidine described in the literature are based primarily on extrapolation from dermatological studies and a limited number of small clinical evaluations. As such, the reliability of conclusions regarding routine cosmetic use is moderate, and further large-scale, long-term studies are needed.

Finally, any new formulation must meet regulatory and manufacturing requirements, including validated analytical methods for content and degradation monitoring, long-term stability testing and demonstration of microbiological safety. Vehicles utilizing novel technologies such as hydrogels or nanocarriers must also undergo dermal irritation and sensitization studies, repeated application safety testing, and scale-up validation to ensure batches reproducibility. These requirements are particularly relevant given the chronic nature of rosacea and the potential for long-term continuous use.

## 10. Nanotechnology and Delivery Systems to Improve Efficacy or Safety of Brimonidine

Ocular administration of brimonidine has short precorneal residence, tear dilution, nasolacrimal drainage and limited corneal penetration—so frequent dosing is typical and adherence can be poor. Platforms that prolong residence and/or enhance penetration can sustain IOP lowering and may reduce systemic exposure and irritation [77]. Similarly, skin application of brimonidine is limited by its low permeability through the stratum corneum, short duration of action, and the potential to cause local adverse reactions such as erythema, burning, or stinging [8]. Moreover, although systemic absorption is generally reduced compared to oral administration, uncontrolled penetration through the skin may still lead to unwanted systemic effects, including hypotension or headache [77]. These drawbacks restrict the overall efficacy and tolerability of brimonidine in dermatological use. Therefore, it is crucial to develop nanotechnology-based delivery systems—such as lipid nanoparticles, nanoemulsions, or vesicular carriers—that can enhance skin penetration, enable sustained and controlled release, reduce local irritation, and minimize systemic exposure, ultimately improving both the safety and therapeutic outcomes of brimonidine in topical applications similarly to ocular formulations [78,79]. However, a review of the current literature has shown that there are significantly more examples of nanotechnology applications in the optimization of formulations and targeted delivery of brimonidine in ophthalmic use. Most nanotechnology studies are preclinical and vary in methodological quality; thus, while they demonstrate clear mechanistic potential, the reliability of their conclusions for clinical dermatology is limited until validated in larger clinical settings.

One example of the use of nanostructured lipid carriers (NLCs) in brimonidine delivery is the study conducted by El-Salamouni et al. [79] who compared NLCs, solid lipid nanoparticles (SLNs), and commercial brimonidine eye drops. NLCs showed favorable properties—small size (~152 nm), high drug entrapment (~84%), and stability for 3 months. They provided 1.23-fold higher corneal permeability than SLNs, localized to the anterior chamber, and achieved the greatest and most sustained IOP reduction (−13.1 mmHg) in rabbits. Overall, the authors showed that NLCs demonstrated clear advantages over SLNs and standard drops, supporting their potential as an improved delivery system for brimonidine [79]. Generally, NLCs represent a promising platform for ocular drug delivery owing to their biodegradability, biocompatibility, and ability to sustain and control drug release while enhancing trans-corneal penetration and ocular bioavailability. Surface modification further enables muco-adhesion, prolonged corneal residence, and improved pharmacokinetics, while incorporation into hybrid systems such as in situ gels or contact lenses expands their therapeutic potential, particularly for ocular surface diseases. Nonetheless, successful clinical translation requires overcoming challenges related to batch reproducibility, colloidal stability, and long-term ocular safety, with quality-by-design strategies providing a rational path toward commercialization [80].

Liposomal formulations of BT have emerged as promising advanced ocular delivery systems, overcoming challenges associated with conventional eye drops such as short residence time, inefficient corneal penetration, and frequent dosing. Cationic-charged liposomes optimized via a Box–Behnken design (using EPCS:DOTAP 1:1) achieved ~150 nm particle size, +30 mV zeta potential, ~55% entrapment efficiency, and sustained drug release reaching ~91% over 12 h, alongside over twofold enhanced ex vivo corneal permeation versus brimonidine solution [81]. In another study, a nano-vesicular/lyophilized formulation dispersed in Carbopol demonstrated a biphasic release profile with reduced burst and sustained intraocular pressure (IOP)-lowering efficacy in rabbits for up to 7.5 h [78].

Apart from liposomes, nanoemulsions and self-nanoemulsifying drug delivery systems (SNEDDS) containing brimonidine have been developed to overcome the limitations of conventional formulations, including short ocular residence time and limited corneal permeability [82]. BT nanoemulsions were developed to improve ocular permeability, onset of action, and therapeutic efficacy in glaucoma management. Using castor oil, Lipoid S75, Lipoid E80, and Poloxamer 68, optimized formulations (BN2, BN3, BN10) achieved nanometric droplet sizes (19–27 nm), narrow polydispersity (PDI ~0.27–0.34), and a favorable zeta potential (−21.3 mV). Multiple studies confirmed compatibility, stability, and reproducible release profiles. These nanoemulsions demonstrated high drug loading, enhanced permeability, prolonged corneal residence time, and sustained drug release, supporting their potential as superior alternatives to conventional ophthalmic formulations for chronic ocular diseases affecting both anterior and posterior segments [83].

In parallel, SNEDDS of brimonidine tartrate was developed using Capmul MCM, Tween 80, and Transcutol HP, optimized via Box–Behnken design. The formulation exhibited a droplet size of ~118 nm, low polydispersity (PDI ~0.25), suitable physiological parameters (pH 6.3, osmolarity ~329 mOsm/L, viscosity ~12 cP), and excellent stability during storage. Ex vivo studies demonstrated a markedly higher apparent permeability coefficient (7.08 × 10^−6^ cm/s) compared to a marketed formulation (3.13 × 10^−6^ cm/s), confirming superior corneal penetration. These findings indicate that SNEDDS represent a promising nanotechnological platform for enhancing ocular bioavailability and therapeutic performance of brimonidine [84]. Together, these systems represent promising nanotechnological strategies for enhancing ocular bioavailability, prolonging therapeutic action, and reducing dosing frequency in glaucoma management.

Another form of nanotechnological device which was used to provide sustained delivery of brimonidine is microsphere. Fedorchak et al. [85] proposed that biodegradable carriers like poly(lactic-co-glycolic acid) (PLGA) microspheres could enable sustained drug release in a formulation resembling conventional eye drops, provided they were paired with a suitable vehicle ensuring retention on the ocular surface. In this context, thermoresponsive hydrogels offer an optimal matrix for embedding drug-loaded microspheres, as they can be administered in liquid form and then reversibly transform into a gel at body temperature, thereby prolonging residence time. The results of these studies showed that a single brimonidine-loaded gel/microsphere (GMS) drop maintained intraocular pressure (IOP) reduction in rabbits for 28 days, achieving effects comparable to twice-daily standard eye drops. The drop remained in the inferior fornix throughout the study, with reduced contralateral eye response, suggesting lower systemic absorption [85]. One more example can be molecularly imprinted HEMA hydrogels for brimonidine increased drug affinity/loading and sustained release compared with non-imprinted lenses. These platforms can maintain therapeutic levels while patients simply wear a lens [86].

While most nanotechnology studies involving brimonidine focus on ocular delivery, there is an innovative dermatological formulation—a nanostructured, thermo-reversible hydrogel—designed to treat skin redness that shows promising enhanced delivery and retention characteristics [73]. Researchers developed thermo-reversible nanostructured supramolecular hydrogels from a cationic bis-imidazolium amphiphile for topical rosacea therapy. Drug incorporation significantly influenced gel properties, with brimonidine tartrate producing the most homogeneous material among the drugs tested [73]. Structural studies showed that drugs were located both within the gel fibers and in the interstitial spaces. Importantly, the hydrogel system provided up to 10-fold faster drug release and up to 20-fold higher skin retention compared with commercial products. In vivo, brimonidine-loaded gels demonstrated rapid and effective erythema reduction [73]. These findings underscore the potential of nanostructured supramolecular hydrogels as advanced topical delivery platforms, particularly for brimonidine in rosacea, as they combine enhanced bioavailability, sustained retention, and superior therapeutic efficacy over conventional formulations.

**Figure 8:**
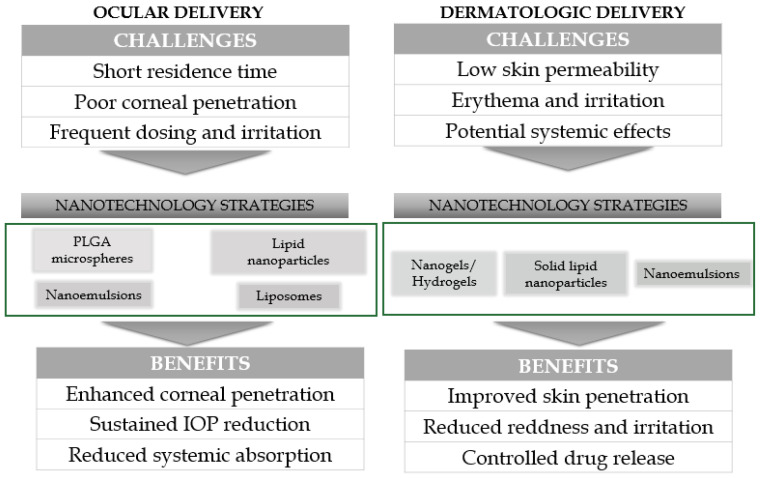
Nanotechnology-Based Strategies for Brimonidine Delivery: Comparison of Ocular and Dermatologic Applications. PLGA—Poly(lactic-co-glycolic acid). IOP—Intraocular Pressure.

Compared with the extensive nanotechnology literature in ophthalmology, where brimonidine has been incorporated into a wide range of nanoparticles, nanoemulsions, nanoliposomes, polymeric micelles, and thermoresponsive gels, dermatology remains significantly underdeveloped. Figure 8 presents comparison of nanotechnology-based strategies for brimonidine delivery (both ocular and topical).

Most nano-formulated brimonidine systems have been designed for glaucoma therapy, aiming to prolong intraocular retention, enhance corneal penetration, or reduce dosing frequency. These formulations operate within a fundamentally different anatomical and pharmacological environment and therefore cannot be directly extrapolated to cutaneous delivery. In contrast, dermatologic nanotechnology studies are scarce and largely preclinical—typically limited to ex vivo permeation studies or rodent skin models, often without standardized endpoints. No human clinical trials have tested nano-brimonidine for erythema reduction, and evidence regarding dermal PK/PD, stability, barrier interaction, or systemic exposure remains fragmentary. Consequently, despite promising theoretical advantages (improved permeation, controlled release, reduced irritation), the actual translational value of nanocarrier-based brimonidine in dermatology remains unverified, and major knowledge gaps persist. Knowledge gaps in nanotechnology for dermatologic brimonidine delivery is summarized in Table 4.

## 11. Reported Derivatives and Analogues of Brimonidine

Brimonidine (UK-14,304) has served as a template for medicinal chemistry exploration of imidazoline–quinoxaline derivatives. The parent structure, 5-bromo-6-(2-imidazolin-2-ylamine)quinoxaline, combines an electron-withdrawing halogen substituent on the quinoxaline core with an imidazoline ring crucial for receptor binding. Over the years, several classes of derivatives have been described in patents and pharmacological studies, focusing on optimization of potency, selectivity, and pharmacokinetic (PK) behavior. Systematic, peer-reviewed structure–activity relationship (SAR) studies dedicated specifically to brimonidine analogues are highly limited in the published literature. As a result, the discussion in this chapter draws on three complementary evidence sources: (i) peer-reviewed medicinal chemistry studies of structurally related α_2_-adrenergic agonists, (ii) experimentally validated receptor-binding data available for imidazoline-based compounds, and (iii) established structural patterns known for imidazoline pharmacophores that influence α_2_/α_1_ selectivity, lipophilicity, and pharmacodynamic behavior. Patent disclosures are included only as supplementary structural examples and are interpreted cautiously, given the absence of peer-reviewed biological validation. Together, these sources provide the most robust and transparent framework currently available for discussing SAR trends relevant to brimonidine.

### 11.1. Halogen- and Ring-Substituted Quinoxalines

Brimonidine contains a bromine atom at the 5-position of the quinoxaline nucleus, a feature that contributes to its lipophilicity and α_2_-adrenergic receptor activity. Although peer-reviewed medicinal chemistry studies on brimonidine analogues are limited, available data on related quinoxaline and imidazoline derivatives—supported by selected experimental observations—provide useful context for interpreting this region of the scaffold.

Patent literature and early exploratory chemistry efforts describe extensive modification at the 5-position, including substitution with chlorine, fluorine, or small alkyl groups such as methyl [87,88]. Variations among halogens were initially explored to examine how size and electronegativity influence receptor affinity and physicochemical properties. Chlorine, being smaller and less lipophilic than bromine, generally maintained α_2_ activity but modestly reduced overall lipophilicity, whereas fluorinated analogues tended to exhibit increased aqueous solubility accompanied by lower receptor affinity [87].

Beyond halogens, small alkyl substituents (e.g., methyl, ethyl) were introduced to probe steric tolerance within the binding pocket. These modifications adjusted lipophilicity in a more subtle manner than halogen exchange and provided insights into size constraints around the 5-position. Some analogues demonstrated improved physicochemical behavior but diminished potency, reinforcing the distinctive contribution of halogen substitution to maintaining high affinity [87].

Further exploration involved ring-disubstituted derivatives, most notably 2,3-dimethyl-5-bromo-quinoxaline analogues [89]. Introduction of methyl groups at the 2,3-positions altered electronic distribution within the quinoxaline scaffold and increased steric bulk near the imidazoline-bearing substituent. These modifications were reported to shift lipophilicity, metabolic stability, and in some cases reduced systemic exposure, a desirable property in the context of minimizing cardiovascular and sedative side effects associated with α_2_ agonists [88]. Such patterns align with broader medicinal chemistry findings in the α_2_-agonist class, where controlled increases in steric bulk can limit systemic penetration without fully compromising target engagement. Consistent with SAR trends observed in clonidine-type α_2_ agonists, halogenation of the aromatic or heteroaromatic core increases lipophilicity and often strengthens receptor binding via enhanced hydrophobic interactions, which helps explain why 5-brominated quinoxalines maintain higher α_2_ affinity compared with non-halogenated or alkyl-only analogues [89].

In summary, the halogen- and ring-substituted quinoxaline derivatives highlight how relatively modest structural changes around the heteroaromatic core can profoundly influence the balance between potency, solubility, and ocular bioavailability. While direct SAR data for brimonidine remain limited, these analogues illustrate the sensitivity of the scaffold to small changes and highlight the importance of carefully balancing lipophilicity with receptor selectivity.

### 11.2. Imidazoline Substituent Variants

Preclinical studies have demonstrated that the compound displays exceptionally high selectivity for α_2_- over α_1_-adrenoceptors, with a selectivity ratio approaching 1000-fold. Moreover, ocular pharmacokinetic investigations confirmed that brimonidine achieves therapeutically relevant concentrations in the posterior segment, including the vitreous and retina, sufficient to activate α_2_ receptors and support proposed neuroprotective actions [90]. Across imidazoline-based α_2_ agonists, preservation of the cyclic imidazoline ring is critical for high α_2_ affinity; replacing it with imidazole, amidine, or open-chain amines consistently lowers potency and α_2_/α_1_ selectivity, confirming the essential pharmacophoric role of this heterocycle.

The imidazoline moiety is a defining pharmacophore in brimonidine and related compounds affecting α_2_ receptors [91]. Peer-reviewed medicinal chemistry studies demonstrate that altering this moiety—by replacing it with imidazole, amidine, N-alkylated imidazolines, or open-chain amines—produces marked shifts in binding affinity and α_2_/α_1_ selectivity [92,93]. For example, medetomidine, one of the best-characterized imidazoline α_2_ agonists, displays an α_2_/α_1_ binding ratio of 5060, compared with 969 for clonidine [93]. Such distinctions highlight the strong dependence of selectivity on the integrity and substitution pattern of the imidazoline/imidazole ring.

In several cases, conformationally constrained analogues displayed altered CNS penetration and reduced sedative effects, underscoring how small structural changes near the imidazoline moiety can modulate both pharmacodynamics and distribution characteristics [94]. Medetomidine and its close analogues preferentially bind the α_2_A receptor subtype, consistent with their sedative and analgesic profile. Platelet assays—representing α_2_A functionality—confirmed strong α_2_A-mediated inhibition of epinephrine-induced aggregation, whereas vascular aorta assays (α_1_ and α_2_B/α_2_C-dominant) revealed markedly lower potency or antagonist behavior in selected analogues [88]. Modifications at the benzylic carbon—especially changes to size, electronegativity, and lipophilicity—caused: large shifts in α_2_ affinity (up to 10–50-fold), altered α_2_A vs. α_2_B/α_2_C selectivity, conversions from agonists to antagonists, depending on ring fusion (1- vs. 2-naphthyl) and the placement of imidazole vs. imidazoline rings [93]. These findings underline the delicate balance between maintaining strong receptor engagement and optimizing distribution characteristics. Peer-reviewed SAR data from medetomidine analogues show that steric rigidity around the benzylic carbon and productive engagement with the α_2_ ‘methyl pocket’ significantly influence subtype selectivity, particularly enhancing α_2_A affinity while diminishing α_2_B/α_2_C activity; disruption of these interactions lowers potency or converts agonists into partial agonists or antagonists [94].

Although direct studies on brimonidine analogues with imidazoline replacements (e.g., imidazole or amidine) and formally quantified CNS penetration or sedative outcomes have not been reported till September 2025, valuable insights can be drawn from work on medetomidine derivatives [93].

In a systematic SAR series, chiral and conformationally rigid medetomidine analogues demonstrated that substituent size, electronegativity, lipophilicity, chirality, and conformational flexibility at the benzylic carbon critically shape affinity and subtype selectivity across α_2_A, α_2_B, and α_2_C receptors [93]. Complementary work identified a hydrophobic “methyl pocket” within the α_2_ receptor, where the benzylic methyl group of medetomidine analogues plays an essential role in anchoring high-affinity binding; removal of this substituent markedly reduced potency [94]. These findings highlight that even subtle steric or electronic modifications around the imidazoline pharmacophore can substantially alter receptor engagement, and, by extension, physicochemical properties such as lipophilicity and polar surface area, which are likely to impact side-effect profiles. While direct brimonidine analogue data remain limited, the mechanistic trends observed across related α_2_ agonist scaffolds provide a scientifically grounded framework for interpreting the potential impact of imidazoline modifications. To complement the SAR discussion, Table 5 summarizes key quantitative data on receptor-binding affinities and α_2_/α_1_ and subtype-selectivity profiles for representative imidazoline- and imidazole-based α_2_ agonists.

### 11.3. Alkylated and Fused Quinoxaline Analogues

Structural diversification around the quinoxaline nucleus has been explored, particularly by introduction of additional alkyl substituents and by creating fused heteroaromatic quinoxaline frameworks. For example, in the U.S. patent Methods for Using (2-imidazolin-2-ylamino) Quinoxaline Derivatives [88], analogues including 2,3-dimethyl-5-bromo-6-(2-imidazolin-2-ylamine)quinoxaline are disclosed. These compounds were designed to modulate physicochemical properties—for instance, lipophilicity (log P), solubility, and metabolic stability—by varying substituents at positions 2, 3, 5 and 6 of the quinoxaline ring [88].

Related patent literature also introduces fused heteroaromatic systems (e.g., tetrahydroquinoxaline derivatives) intended to further modulate both physicochemical and pharmacokinetic behavior by altering ring rigidity and electronic distribution [95]. While several of these structures demonstrated favorable in vitro attributes, few appear to have advanced beyond early preclinical evaluation, and publicly available data on their in vivo pharmacokinetics, toxicity, or tolerability remain limited. This underscores the largely exploratory nature of these scaffold modifications and the current absence of peer-reviewed evidence confirming their translational potential.

### 11.4. Related α_2_-Adrenergic Agonists with Alternative Scaffolds

In parallel with the development of direct analogues of brimonidine, structurally distinct imidazoline derivatives such as clonidine, apraclonidine, and dexmedetomidine have been studied extensively as α_2_-adrenergic agonists. Although these agents are not derivatives of brimonidine per se, they furnish critical pharmacological context: by comparing their aromatic scaffolds (e.g., differences in halogen substitution on phenyl rings, presence or absence of additional ring substituents), imidazoline ring substitution, lipophilicity, and ability to cross the blood–brain barrier, one can discern how structural variation modulates α_2_ vs. α_1_ selectivity, subtype preferences (α_2A_, α_2B_, α_2C_), central vs. peripheral effects, efficacy, and side-effect profiles [91,96].

For instance, clonidine, one of the earliest imidazoline-type α_2_ agonists, contains a dichlorophenyl-imidazoline structure with relatively modest α_2_:α_1_ selectivity (approximately 220:1) compared to newer agents, permitting greater α_1_-mediated side effects (e.g., vascular constriction) as well as more central actions [91,97].

Apraclonidine is a halogenated derivative of clonidine (*p*-aminoclonidine) designed to retain α_2_-agonism but with altered pharmacokinetics: it is more hydrophilic with lower blood–brain barrier penetration, has a somewhat lower α_2_:α_1_ selectivity than brimonidine, and mainly acts locally in the eye to reduce aqueous humor production [90,98]. Its side-effect profile is different, in part because of its reduced central penetration [99].

Dexmedetomidine (the dextro-isomer of medetomidine) exemplifies a scaffold that achieves very high α_2_ vs. α_1_ selectivity (on the order of ~1600:1), much greater than clonidine’s, leading to potent sedative, analgesic, and anxiolytic effects with relatively fewer α_1_-mediated adverse effects [100,101]. It also has increased lipophilicity and optimized substitution that favor central nervous system access when given systemically. Its subtype binding (α_2A_ in particular) and its pharmacokinetic profile further distinguish it [97].

In summary, brimonidine, has served as a key template in developing α_2_-adrenergic agonists, although peer-reviewed SAR studies on its direct analogues remain limited. Modifications around the quinoxaline ring—especially halogen substitution at the 5-position and alkylation at the 2,3-positions—revealed how small structural changes strongly affect potency, solubility, receptor selectivity, and ocular bioavailability. The imidazoline ring is the defining pharmacophore, and peer-reviewed studies of medetomidine analogues show how its integrity, steric environment, and interaction with key hydrophobic motifs critically shape α_2_ affinity, α_2_/α_1_ selectivity, and CNS penetration. Additional strategies, including further alkylation and fused quinoxaline scaffolds, aimed to optimize lipophilicity, solubility, and pharmacokinetics, though most remained at the preclinical stage. In parallel, structurally distinct imidazoline α_2_-agonists—such as clonidine, apraclonidine, and dexmedetomidine—provide experimentally validated SAR benchmarks; their differences in halogenation patterns, lipophilicity, and subtype selectivity illustrate how scaffold variation governs receptor engagement and distribution. Together, these data help contextualize brimonidine-related SAR despite the current scarcity of dedicated peer-reviewed studies.

## 12. Future Perspectives on the Application of Brimonidine in Dermatology

Despite its established role in the management of rosacea-associated erythema [8], brimonidine’s pharmacological potential in dermatology is far from fully realized. Future research is expected to expand its clinical applications by leveraging its vasoconstrictive, anti-inflammatory, and neurovascular modulatory properties in new therapeutic contexts.

Recent preclinical evidence suggests that brimonidine may modulate cutaneous immune responses and oxidative stress, indicating possible benefits in inflammatory dermatoses beyond rosacea, such as seborrheic dermatitis, atopic dermatitis, or acne-associated erythema [40,47]. Its demonstrated ability to stabilize the endothelial barrier and suppress leukocyte migration opens opportunities for use in acute inflammatory conditions, photodermatoses, or as a supportive agent in barrier-repair formulations [48]. Despite the growing number of dermatology-focused studies on brimonidine, the current evidence base is limited by several important methodological weaknesses. Many clinical investigations—particularly those assessing post-procedural erythema, alcohol flushing syndrome, telangiectasia, hemostasis, or combination regimens—are characterized by small sample sizes, single-center recruitment, split-face or open-label designs, and short follow-up periods. These studies offer valuable preliminary signals of efficacy but lack the statistical power and external validity required to draw definitive conclusions or establish comparative effectiveness versus other α-adrenergic agonists (e.g., oxymetazoline). Long-term controlled data remain scarce, and the majority of safety conclusions are extrapolated from short-duration RCTs or post-marketing observations rather than rigorously designed prospective safety studies.

Mechanistic understanding also remains incomplete. While vasoconstriction is well documented, the relative contribution of anti-inflammatory, neurovascular, and endothelial-stabilizing actions is insufficiently defined in human skin. No clinical trials incorporate mechanistic biomarkers, neuropeptide assessments, vascular imaging, or quantification of mast-cell or TRP-channel activity. Consequently, predictors of treatment response and the pathophysiology of paradoxical or rebound erythema remain uncertain. Dermal pharmacokinetic/pharmacodynamic (PK/PD) profiles are likewise unknown: cutaneous receptor occupancy, depth-dependent distribution, concentration–effect relationships, and systemic exposure under maximal-use or barrier-impaired conditions have not been systematically evaluated.

Technological advances will play a pivotal role in broadening the dermatologic utility of brimonidine. Novel drug delivery platforms, including nanostructured lipid carriers, supramolecular hydrogels, and microemulsions, have the potential to optimize topical bioavailability, prolong therapeutic action, and minimize adverse effects such as rebound erythema [78,79]. As far as the authors are concerned, integration of brimonidine into multi-component formulations—particularly with agents such as oxymetazoline, tranexamic acid, or anti-inflammatory peptides—could further enhance its safety and clinical outcomes. The design of sustained-release systems tailored for controlled dermal absorption represents a promising direction for improving patient adherence and expanding indications.

Another field that should be further developed is the design and synthesis of new brimonidine analogues with high selectivity for α-adrenergic receptors located in cutaneous blood vessels. Exploration of brimonidine analogues or derivatives with modified receptor selectivity and optimized physicochemical properties could yield next-generation topical agents with broader efficacy and reduced side-effect profiles.

In summary, the future of brimonidine in dermatology lies in multidisciplinary innovation that bridges pharmacology, material science, and molecular dermatology. Through targeted delivery, mechanistic elucidation, and development of novel analogues, brimonidine may evolve from a niche treatment for rosacea to a versatile therapeutic platform addressing vascular, inflammatory, and cosmetic skin concerns.

## 13. Conclusions

Brimonidine has become an important therapeutic option in dermatology, particularly for managing persistent facial erythema in rosacea. Its rapid and reversible vasoconstrictive action, combined with anti-inflammatory and endothelial-stabilizing effects, effectively reduces redness and improves patient satisfaction.

Beyond rosacea, growing evidence supports the use of brimonidine in alcohol flushing syndrome, post-procedural erythema, and as a topical hemostatic agent in dermatologic surgery. The compound demonstrates good safety and tolerability with minimal systemic absorption, though awareness of potential adverse effects such as rebound erythema remains essential.

Advances in formulation technology, including nanostructured lipid carriers and supramolecular hydrogels, offer promising strategies to enhance skin penetration, extend duration of action, and reduce local irritation. In parallel, the design of novel brimonidine analogues with increased α_2_-adrenergic selectivity for cutaneous vessels could further optimize therapeutic outcomes.

Overall, brimonidine represents a versatile agent and promising scaffold for future drug development. Continued interdisciplinary research integrating pharmacology, formulation science, and molecular design may expand its dermatologic and cosmetic applications in the coming years.

## Figures and Tables

**Table 1:** Pharmacokinetic properties of brimonidine after topical ocular administration: comparison across formulations and animal models.

Model	Formulation	C_max_ (Aqueous Humor)	T_max_ (AH)	AUC/MRT	Half-Life/Notes	Ref.
Albino vs. pigmented rabbits (single 0.5% dose, 35 µL drop)	Topical (^14^C-brimonidine)	Albino: 2.16 ± 0.75 µg/mL Pigmented: 1.52 ± 0.38 µg/mL	~0.67 h	-	AH half-life ~1 h (initial); Pigmented iris-ciliary body terminal half-life ~160 h	[28]
ISG vs. eye drops (anterior chamber PK by micro-dialysis)	Ion-sensitive in situ gel (BRT-ISG) vs. conventional drops	Drops: 3.06 ± 1.42 mg/L ISG: 8.16 ± 2.62 mg/L	Drops: 60 ± 21 min ISG: 93 ± 29 min	AUC_0–t_ (min·mg/L): Drops: 412.6 ± 204.3 ISG: 1397.1 ± 444.6 MRT_0–t_ (min): Drops: 110.3 ± 18.2 ISG: 140.1 ± 17.5	Drops: 64.99 ± 19.18 min ISG: 75.60 ± 17.66 min	[29]
Rabbits, topical with/without timolol (plasma PK)	0.1% brimonidine single vs. fixed-combination	Plasma C_max_: Combination 1190 ± 230 pg/mL; Alone 947 ± 70 pg/mL	Combination: 0.33 h; Alone: 0.43 h	Plasma AUC_0–4_: Combination: 1540 ± 100 pg·h/mL; Alone: 1380 ± 100 pg·h/mL	-	[30]

Abbreviations: AH—Aqueous Humor; AUC—Area Under the Curve (concentration × time); BRT-ISG—Brimonidine Tartrate Ion-Sensitive Gel: sustained-release ocular formulation forming a gel after instillation; C_max_—Maximum Concentration; ISG—In Situ Gel: formulation that undergoes sol-to-gel transition under physiological conditions; MRT—Mean Residence Time, PK—Pharmacokinetics; T_max_—Time to Maximum Concentration.

**Table 2:** Critical appraisal of key clinical studies evaluating topical brimonidine in dermatology.

Study (Author, Year)	Indication	Design & Sample Size	Strengths	Limitations	Reliability	Ref.
Fowler et al., 2013/2014	Rosacea-related persistent erythema	Two randomized, double-blind, vehicle-controlled trials; *n* ≈ 553	Large sample size; robust RCT design; validated clinician & patient scales; clear dose—response data	Short duration (4 weeks); lack of long-term outcomes; no comparator with other α-agonists	+++	[50]
Micali et al., 2014	Rosacea (erythema + telangiectasia)	Combination Nd:YAG + brimonidine; uncontrolled combination design; *n* = 18	Addresses multi-modal therapy; uses Investigator’s Global Assessment	Very small cohort; no control group for combination effect; no blinding	++	[52]
Wesley Yu et al., 2020	Alcohol flushing syndrome	Randomized, double-blind, split-face; *n* = 20	Within-subject control reduces variability; strong effect size	Very small sample; single ethnic group (East Asian); short follow-up	++	[55]
Chen et al., 2018	Intraoperative bleeding in Mohs micrographic surgery	Randomized pilot; *n* = 24 (14 treated)	Novel indication; objective bleeding measurement; clinically meaningful outcome	Pilot nature; underpowered; no blinding reported; surrogate endpoint	+	[56]
Lipner et al., 2015	Hemostasis in nail surgery	Case series; *n* = 10	Real-world evidence; consistent hemostatic effect	No controls; no randomization; potential reporting bias	+	[57]
Moore et al., 2015	Rosacea (persistent erythema)	Prospective, open-label extension; *n* = 449	Long-term safety data (up to 1 year)	No control group; open-label; reports safety but not comparative efficacy	++	[59]
Vissing et al., 2018	Post-IPL erythema and discomfort	Randomized, single blinded, split-face; *n* = 19	Well-controlled within-subject design; objective reduction in erythema	Small cohort; short-term outcomes; single-center	++	[58]
Agamia et al., 2022	Persistent post-acne erythema	Split-face, placebo-controlled; *n* = 30	Tests combination therapy; significant erythema reduction	Hard to isolate brimonidine’s contribution; small sample; short follow-up	++	[53]
Almebayadh, 2019	Ulcerated infantile hemangioma	Case, *n* = 2	Novel use, rapid healing	Extremely small; uncontrolled; safety unknown	None	[60]

+ low reliability; ++ average reliability of the study; +++ high reliability.

**Table 3:** Investigational Off-Label Uses of Brimonidine in Aesthetic Dermatology.

Target Cosmetic Concern	Mechanism	Type of Formulation	Expected Effect	Potential Use
Transient erythema (flushing)	Vasoconstriction via α_2_-receptors	Topical gel/cream	Rapid reduction of redness	Sensitive skin, rosacea-prone skin
Post-procedure redness (e.g., laser, peel)	Reduced microvascular permeability	Cooling gel, hydrogel patch	Soothing and calming	Dermatological after-care
Daily tone correction	Mild vasoconstrictive + anti-inflammatory activity	Cream or serum	Even skin tone, less visible vessels	Cosmetic skincare
Irritation-induced redness	Anti-inflammatory modulation	Serum or emulsion with brimonidine derivative	Decreased inflammatory redness	After sun or after shaving
Couperose-prone skin	Improved microvascular stability	Long-term use cream	Less visible telangiectasias	Mature or sensitive skin

**Table 4:** Knowledge Gaps in Nanotechnology-Based Brimonidine Delivery for Dermatologic Applications.

Topic	What Is Known (Evidence)	Knowledge Gaps (Unknowns)	Implications for Future Research
Evidence base distribution	Brimonidine nanocarriers (nanoparticles, liposomes, nanoemulsions, hydrogels) are well-studied in ophthalmology.	Dermatology-specific nano-formulations are extremely limited, mostly preclinical; no human trials.	Develop dermal-focused nano-formulations; avoid extrapolating ocular data to skin.
Dermal penetration & biodistribution	Single rodent data show improved permeation vs. conventional gels.	No human dermal PK/PD data (local concentration, retention, release kinetics).	Conduct dermal micro-dialysis, tape-stripping studies, PK/PD modelling.
Clinical efficacy	Theoretical advantages: enhanced bioavailability, sustained release, improved stability.	No clinical evidence that nano-brimonidine improves erythema, duration, or tolerability over commercial gel.	Phase I RCTs comparing nano-formulations vs. 0.33% gel using objective erythema metrics.
Comparisons across nanocarrier types	Many carrier classes exist in literature	No head-to-head comparisons in dermatology (e.g., PLGA vs. nanoemulsion vs. nanoliposome) using unified protocols.	Systematic comparative studies with standardized endpoints
Systemic absorption & safety	Nano-delivery can change biodistribution in other fields.	Unknown how nanocarriers alter systemic exposure in skin.	Maximal-use systemic PK studies, barrier-impaired skin models, safety monitoring.
Irritation & immunologic safety	Limited cytotoxicity data from in vitro assays.	No long-term irritation, sensitization, or immunologic safety data for dermal nano-brimonidine.	In vivo irritation/sensitization studies, repeated-dose dermal toxicity.

**Table 5:** Receptor-binding affinities and subtype-selectivity data for imidazoline/imidazole α_2_-agonist analogues.

Compound or Analogue Class	Key Structural Feature	α_2_ Affinity (Representative Data)	α_2_/α_1_ Selectivity	Reference
Clonidine	Imidazoline ring; dichlorophenyl	High α_2_ affinity; standard reference agonist	~969	[93]
Medetomidine	Imidazoline with benzylic methyl group	Very high affinity for α_2_	~5060 (≈5× clonidine).	[93]
Medetomidine analogues (rigid, chiral, fused-ring variants)	Benzylic substitutions; conformational restriction	Affinity varies 10–50× depending on substitution	Selectivity shifts widely based on steric/electronic changes	[93]
Benzylic-desmethyl medetomidine analogues	Removal of benzylic methyl	Markedly reduced α_2_ affinity	Strong drop in α_2_ selectivity	[93]
Imidazole analogues (imidazoline → imidazole replacement)	Imidazole ring instead of imidazoline	Typically reduced α_2_ affinity	Lower α_2_/α_1_ selectivity	[93]
Amidine analogues	Imidazoline → amidine	Variable affinity; often lower than imidazoline	Reduced selectivity	[94]
Open-chain amines	Loss of cyclic imidazoline	Strongly reduced affinity	Poor selectivity	[94]

## Data Availability

Not applicable.
